# Urinary Electrophoretograms Performed by Capillary Electrophoresis: Comparison between Dogs and Cats

**DOI:** 10.3390/gels9070544

**Published:** 2023-07-04

**Authors:** Laura Gil, Martyna Wsol, Salceda Fernández-Barredo, Paula Fátima Navarro

**Affiliations:** 1 Department of Veterinary Medicine and Surgery, Universidad Católica de Valencia San Vicente Mártir, 46001 Valencia, Spain; laura.gil@ucv.es; 2 Facultad de Veterinaria, Universidad Católica de Valencia San Vicente Mártir, 46001 Valencia, Spain; martyna.mw9@gmail.com; 3 CEDIVET (Centro Diagnóstico Veterinario), 46988 Valencia, Spain; salcedaf@gmail.com

**Keywords:** electrophoresis, feline, proteinuria, renal, urine

## Abstract

Electrophoresis of urine to evaluate different urinary proteins has been used in recent years in veterinary medicine, as it can be a useful laboratory tool in the early detection of kidney damage. However, urinary capillary electrophoresis (UCE) has not been reported in healthy cats. In healthy dogs, reference intervals have been established and can be compared with pathological samples as these provide an easily interpretable pattern. The electrophoretogram in this study is divided into five fractions (F1–F5) by serum (albumin; alpha_1_-globulin; alpha_2_-globulin; beta-globulin; and gamma-globulin). Urine samples from 14 healthy cats were obtained by eco-guided cystocentesis. UCE was run in all samples and compared to 123 dog electrophoretograms from a previously published study. Fraction 2 (alpha_1_-globulin) was statistically decreased in cats (G_1_) compared to dogs (G_2_). Fraction 4 (beta-globulin) was statistically augmented in cats compared to the canine population (G_2_). Fraction 5 (gamma-globulin) was statistically decreased in cats (G_1_) compared to dogs (G_2_). No statistical correlation was found between each cat’s serum and urinary fractions. The results of the present study suggest that UCE patterns in cats are similar to the ones described in dogs. UCE can be a non-invasive new diagnostic tool in cats as pathological patterns can be compared to normal ones.

## 1. Introduction

Electrophoresis is a laboratory technique based on separating molecules according to their charge, molecular weight, and structure when subjected to an electric field [1]. The biomolecules migrate from the cathode or anode, resulting in an electrophoretic pattern. Electrophoresis can be classified as zonal or capillary. Zonal electrophoresis requires a solid support such as a porous gel, usually a polymer (agarose or acrylamide); conversely, capillary electrophoresis (CE) uses silica capillaries to separate the biomolecules [2,3,4].

Serum reference interval proteins in cats performed by gel electrophoresis are 29.00–46.70 for albumin, which is higher compared to dogs (27.20–44.90); 2.08–4.99 for alpha_1_-globulin, which is lower than in dogs (3.38–9.09); 2.94–10.25 for alpha_2_-globulin, which is similar to that found in dogs (2.37–9.58); 3.05–9.4 for beta-globulin, which is lower than in dogs (5.07–16.51); and 4.33–21–40 for gamma-globulin, which is higher in cats than in dogs (2.26–10.70) [5,6].

The urinary electrophoretogram obtained is commonly divided into five different fractions from low to high molecular weight and charge, as is usually done in serum: fraction 1 migrates in the albumin zone; fraction 2 in the alpha_1_-globulins zone; fraction 3 in the alpha_2_-globulins zone; fraction 4 in beta-globulins; and fraction 5 in the gamma-globulins zone [6,7,8].

Analysis of human urinary proteins by CE has been proven a valid method for detecting the presence of characteristic electrophoretograms in metabolic, inflammatory, infectious, neoplastic, and immune-mediated diseases [9,10,11]. Recently, capillary electrophoresis in combination with contactless conductivity detection has been proven to be an excellent technique for the detection of biologically active substances, such as low-molecular-weight proteins [12]. In veterinary medicine, gel electrophoresis is the most widely used technique for the study of proteinuria and renal disorders. Several studies have used sodium dodecyl sulfate agarose–polyacrylamide gel electrophoresis (SDS-AGE; SDS-PAGE) that correlates the patterns obtained with different diseases [13,14,15,16,17,18]. This technique is considered the gold standard for the characterization of the proteins in urine as it separates proteins based on molecular weight, allowing the location of the damage to be identified in either the glomerulus, the tubule, or both [19]. Before electrophoresis, urine needs to be concentrated by ultrafiltration columns. Samples can be applied in each well and run at 272 V and 20 °C; once the gel dries, each well is stained with a solution with amido Schwarz in acetic acid. Finally, when it has dried at 75 °C for 8 min, the gel can be scanned [9].

Glomerular proteinuria occurs when the selective permeability of the glomerular basement membrane is altered, and it is characterized by the excretion of medium-molecular-weight (40–69 kDa) and high-molecular-weight molecules (≥70 kDa) [19,20,21]. Damage to the glomerulus results from the formation and deposition of immune complexes, causing lesions such as membranous, membranoproliferative, glomerular sclerosis, or amyloidosis. In addition, alterations at the glomerular level can develop secondary to acquired systemic processes such as neoplasms, infectious diseases, chronic inflammatory diseases, heart disease, or secondary to endocrine disorders [22]. This type of glomerular proteinuria can be classified according to the molecular weight of the protein bands observed in the gels, so protein bands ≥ 40 kDa could be considered a glomerular electrophoretic pattern [23].

Tubular proteinuria is characterized by low-molecular-weight molecules (<40 kDa) in the urine. Usually, these proteins freely cross the glomerular filtration membrane and are reabsorbed by the proximal tubule. However, when there is a tubular injury, protein reabsorption is affected, generating urinary excretion [19]. The causes of this tubular proteinuria are acute tubular necrosis and Fanconi syndrome, among others [24]. This type of tubular proteinuria can be classified according to the molecular weight, so protein bands <40 kDa could be considered a tubular electrophoretic pattern [19].

Finally, mixed proteinuria occurs when glomerular and tubular lesions develop simultaneously, showing low-, medium-, or high-molecular-weight proteins in the urine [19,20]. This type of tubular proteinuria can be classified according to the molecular weight of the protein bands, so low-, medium-, or high-molecular-weight bands could be considered mixed electrophoretic patterns [23].

Regarding the study of kidney disease, gel electrophoresis has been proven to be a sensitive technique for diagnosing tubulointerstitial disease but with lower specificity in differentiating between glomerular disorders [15,17].

UCE is a technique still under study, although interval references for the canine species have been determined [8]. Moreover, electrophoretogram patterns for dogs with azotemia due to *Leishmania infantum* (*L. infantum*) or chronic kidney disease not associated with Leishmaniosis, compared to healthy ones, have been described with UCE [25].

Although quantitative proteinuria can only be assessed by the urine protein:creatinine (UPC) ratio, urine electrophoresis can be used as a semi-quantitative method and provides different patterns that can be related to health or renal and extrarenal diseases [16,17,18,25,26]. In UCE, the proteins in each fraction cannot be identified by their molecular weight as in gel electrophoresis, which may be a disadvantage in evaluating glomerular or tubular proteinuria [4]. The combination of the UCE technique with mass spectrophotometry or immunofixation could assess which proteins are excreted in each fraction of the urinary electrophoretogram so that these proteins could be used as urinary biomarkers for the diagnosis and monitoring of different pathologies [25].

The aim of this study was to use urinary capillary electrophoresis in healthy cats for the first time and a comparison of urinary electrophoretograms in healthy dogs and cats to assess the difference between the two species.

## 2. Results and Discussion

### 2.1. Cat Study Population (G_1_)

A total of 14 samples from healthy cats were included, and 14 samples were eliminated as they did not meet the inclusion criteria; the median age was 3.75 ± 2.66; 10 females and 4 males were included; 9 neutered females and 1 intact female were included; and 2 castrated and 2 intact males were included. No significant laboratory abnormalities were found in this group. All UPCs ratios were <0.2 (non-proteinuric). All cats tested negative for FeLV antigen and FIV antibody.

### 2.2. Dog Study Population (G_2_)

Data from a previous study of 123 samples from healthy dogs were included. The median age was 6.64 ± 3.04, and the gender distribution was 54 males and 69 females. No significant laboratory abnormalities were found in this group, except in serum protein electrophoresis in dogs older than seven years, where a polyclonal increase in the gamma-globulin fraction (RI: 6–12%) was found. All dogs tested negative for *L. infantum* antigen. All UPC ratios included were <0.5.

### 2.3. Protein Fractions

**Figure 1:**
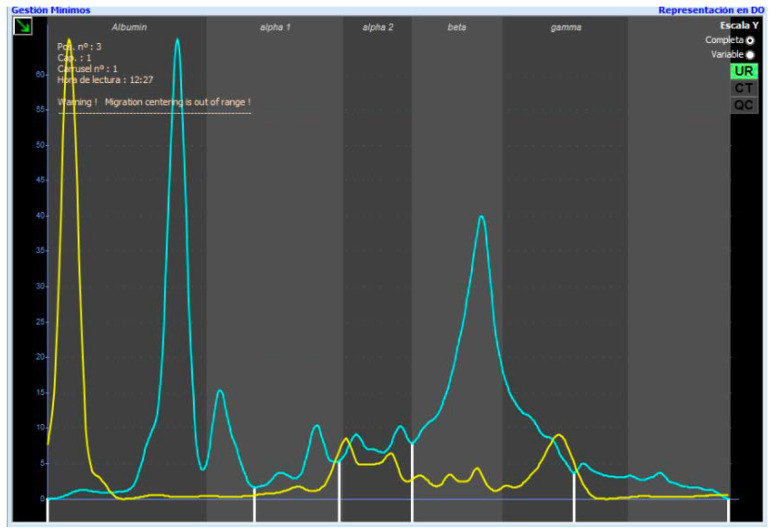
Overly healthy cat serum diluted 1:49 with the urinary electrophoretogram of a healthy cat with the five different fractions. The yellow line represents the feline diluted serum and the blue line is the electrophoretic pattern of the studied urine.

The electrophoretic urine pattern obtained for each cat was divided into five protein fractions: F1 or albumin; F2 or alpha_1_-globulin; F3 or alpha_2_-globulin; F3 or beta-globulin; and F5 or gamma-globulin. After the division, the percentage of each fraction was obtained. These fractions were determined according to a diluted serum from a healthy cat superposed on every urine sample (Figure 1). Although the fractions do not migrate at the same point, they can be used as a guide, as the different fractions have similar characteristics. In some studies, albumin in urine tends to migrate near the anode compared to serum in CE, which could be the reason why in urine, albumin migrates later than in serum (Figure 1) [27,28].

### 2.4. Comparison between the Urinary Electrophoretograms of Dogs and Cats

A one-way ANOVA was performed to compare the percentages obtained after the separation of the five different fractions between G_1_ (*n* = 14) and G_2_ (*n* = 123) in the electrophoretogram (Table 1).

- Albumin (F1): one outlier was found and eliminated from G_1_ (*n* = 13). No statistical significance was found between G_1_ and G_2_.
- Alpha_1_-globulin (F2): two outliers were found and eliminated from G_2_ (*n* = 121). F2 was significantly decreased in G_1_ compared to G_2_ (*p* = 0.00).
- Alpha_2_-globulin (F3): two outliers were found and eliminated from G_2_ (*n* = 121). No statistical significance was found between G_1_ and G_2_.
- Beta-globulin (F4): two outliers were found and eliminated from G_1_ (*n* = 11). F4 was significantly higher in G_1_ than in G_2_ (*p* = 0.01).
- Gamma-globulin (F5): six outliers were found and eliminated from G_2_ (*n* = 117). F5 was significantly lower in G_1_ than in G_2_ (*p* = 0.00).

In the present study, F1 was not statistically significant, although the mean values showed a downward trend compared to dogs. In a study with high-resolution electrophoresis, low albumin concentrations were detected in cats’ urine [29]. Therefore, this fraction could contain a lower amount of this protein in the case of felines, which, considering that it is the most abundant protein in the urine of healthy animals, may be associated with lower UPC values in cats compared to dogs [30,31,32]. In this study, all cats and dogs were healthy, so protein excretion was expected to be low [33]. Interpretation of these results suggests that, in general, cats may have lower urinary albumin levels compared to dogs. This could be due to physiological differences between species or to factors specific to the sample or method of analysis used in the present study.

F2 was significantly lower in G_1_ than in G_2_. Some studies have pointed out that in F2, anti-inflammatory proteins are excreted; also, a decrease in the proteins in this fraction has been seen when some treatments, such as vaccines that stimulate the immune system, are administrated [34,35]. However, it is essential to note that the present study did not provide data on preventive care, including vaccinations or deworming treatments, for cats and dogs. The lower levels of anti-inflammatory proteins in the urine of cats compared to dogs in this study can be explained, indicating a lower excretion of these anti-inflammatory proteins in cats. Additionally, the absence of preventive care data, such as vaccination history, limits the ability to establish a direct relationship between specific treatments and the observed differences in the F2 fraction. Future studies considering preventive care interventions and larger sample sizes are necessary to further investigate the impact of vaccinations and other treatments on the F2 fraction of the urinary electrophoretogram in cats and dogs.

F3 was not found to be statistically significant. There are no studies in cats about the excretion of proteins in this fraction. In serum, this fraction increases in cases of inflammatory disease or nephrotic syndrome, which can be related to the fact that healthy cats and dogs do not excrete many proteins in this fraction [35,36]. To further understand the excretion patterns of proteins in the F3 fraction of cat urine, future research endeavors could investigate urinary protein profiles in cats with specific diseases or conditions associated with altered protein excretion.

F4 was significantly higher in G_1_ compared to G_2_. Previous studies that performed gel electrophoresis in urine from healthy individuals have found that this fraction contains proteins related to the defense of the urinary tract. According to previous studies, the Tamm–Horsfall protein or uromodulin is usually excreted in healthy individuals due to its protective properties against lower urinary tract infections [8,29,37]. The high-resolution electrophoresis technique detected that one of the major proteins in the urine of healthy cats was uromodulin [29], so this protein could be excreted in a higher proportion in the F4 urinary fraction in the feline species. A negative correlation has also been observed between the amount of uromodulin in the urine [37,38,39] and the progression of renal pathology in dogs.

F5 was found to be significantly lower in G_1_ compared to G_2_. In serum, this fraction is higher in cats with feline infectious peritonitis, lymphoma, or chronic gingivostomatitis [5]. It is important to note that these studies involve cats with specific diseases, while the present study only included healthy animals. Additionally, it has been observed that cats generally excrete fewer proteins in urine compared to dogs under normal physiological conditions. This difference may be attributed to species-specific renal physiology and protein metabolism variations. Cats have a lower glomerular filtration rate (GFR) than dogs, which could affect the excretion of proteins in the urine [30]. To gain a more comprehensive understanding of the significance of the lower F5 fraction in cats, further research involving larger sample sizes, including diseased cats, and considering factors such as age, breed, and health status, would be valuable. Investigating the specific composition and functions of the protein content in the F5 urinary fraction in cats could provide insights into their role in feline health and disease.

### 2.5. Comparison between Urinary and Serum Electrophoretograms in the Cat Population (G_1_)

Linear regression was performed to predict the relationship between serum and urinary fractions in G_1_ (Table 2).

No correlation was found between the changes in the different electrophoretic fractions of serum and urine of the cat population. The fractions analyzed included albumin (F1), alpha1-globulin (F2), alpha2-globulin (F3), beta-globulin (F4), and gamma-globulin (F5).

The lack of correlation between the serum and urinary fractions in G_1_ may be attributed to the healthy status of the population and the expected low protein excretion in urine [40]. Previous studies have also reported a lack of association between serum and urine fractions in healthy dogs or dogs with kidney disease, where hypoalbuminemia is commonly observed [25].

The limitations of the present study include the limited sample population of feline patients, as well as the need for more data collection that may be important in urinary electrophoretic patterns, such as the vaccination and deworming status of the individuals included in the study.

Another limitation would be the difficulty involved in comparing the two electrophoresis techniques (capillary vs. gel) used to evaluate proteinuria since these techniques use different physical methods to separate the proteins. Urinary capillary electrophoresis is a technique that allows the separation and quantification of proteins in urine by applying an electric field through a silica capillary. It has proven to be a sensitive and specific tool for detecting and characterizing different protein fractions in urine, including albumin, globulins, and other low-molecular-weight proteins [2,3,4]. Urinary capillary electrophoresis has been used in studies to assess proteinuria in dogs, providing information on protein profiles and possible alterations associated with renal and systemic diseases [25]. Instead, agarose gel electrophoresis is a widely used technique for separating proteins in biological samples. In evaluating proteinuria, agarose gel electrophoresis can be applied to the urine to identify and quantify different protein fractions, such as albumin and globulins. This technique allows good resolution of protein bands and has been used in studies to assess proteinuria in dogs and cats [13,14,15,16,17,18,19]. Both methods have advantages and disadvantages in evaluating proteinuria in dogs and cats. Urinary capillary electrophoresis offers greater sensitivity and the possibility of analyzing smaller samples. In addition, it allows a faster separation of proteins and a better characterization of protein profiles. On the other hand, agarose gel electrophoresis is widely accessible, cheaper, and offers a high resolution of protein bands, which facilitates the identification of specific proteins [2,3,4,28,29]. Therefore, urinary capillary electrophoresis and agarose gel electrophoresis are helpful techniques for evaluating proteinuria in dogs and cats. The choice of method will depend on factors such as the sensitivity required, the availability of equipment and resources, as well as the experience of the technical staff.

## 3. Conclusions

Urinary electrophoresis in the feline species could be a new diagnostic and monitoring tool, but additional studies with a larger population are necessary to obtain reference intervals. Comparison between dogs and cats shows that F2 (alpha_1_-globulin) and F5 (gamma-globulin) appear to be significantly lower in cats, while F4 (betaglobulin) is found to be significantly higher. Changes in serum electrophoretogram are unrelated to a higher or lower number of proteins in the different fractions of the urinary electrophoretogram.

## 4. Materials and Methods

### 4.1. Study Population and Sample Collection

The study population was located in Valencia. Twenty-eight samples from apparently healthy cats were included in the study from 2019 to 2022. Data from 123 samples from healthy dogs from a previous study were included (G_2_). Healthy cats had to meet the following criteria: normal anamnesis and physical examination, without medications at the time of the study, and blood and urinary parameters within reference limits for laboratory results. Inclusion criteria such as age, sex, breed, or reproductive status were irrelevant in this study, and cats were randomly included with regard to these.

Blood samples were collected from the jugular vein. A minimum of 2 mL was collected from each cat. Blood was preserved until analysis in a 0.5 mL EDTA tube (Aquisel) and a 1 mL tiger-top tube for serum collection (Aquisel). The analysis included a complete blood count (Celltac Alpha VET MEK-6550; Nihon) and blood smear evaluation. Biochemical analyses included BUN, creatinine, alanine aminotransferase, and total serum proteins (CS 300 analyzer; Diriu). Serum electrophoretograms were performed by CE (Minicap; Sebia). The serum of all cats was tested for FeLV antigen and FIV antibodies.

Urinary samples were collected by eco-guided cystocentesis with an 5 mL syringe and a 0.7 × 40 mm needle. A minimum of 4 mL was required for the study. Complete urinalysis was performed after the extraction. The following parameters were evaluated with fresh urine: pH, glucose, ketones, bilirubin, and hemoglobin (LabStrip u11 Plus; 77 Elektronika); specific gravity with a refractometer (Optica Ponteranica); microscopic fresh and stained sediment (binocular microscope DM 500; Leika); total urine protein and creatinine ratio (CS 300 analyzer; Diriu); and urine culture in a specific chromogenic medium (Chromagar orientation medium; Becton Dickinson). Urine samples were centrifuged at 804× *g* for 10 min (Centrifuge 2650; Nahita), and the supernatants were stored in 4 microcentrifuge tubes with 1 mL aliquots at −20° before dialysis.

### 4.2. Urinary Capillary Electrophoresis

UCE was performed according to the standardized method described by Navarro et al. (2021). Urine was dialyzed before CE to eliminate salts and compounds that could interfere with the wavelength used for reading and cause artifact peaks. Urinary supernatant (4 mL) from each cat was thawed at room temperature and centrifuged at 1.609× *g* for 10 min. The resulting supernatant was transferred to a 4 mL dialysate column with a filter (Vivaspin Turbo 4 10.000 MWCO; Sartorius, Göttingen, Germany). Dialysate columns with urine were centrifuged at 1.878× *g* for 25 min. The urine that remained at the bottom of the container was discarded. A washing solution was prepared, adding 50% of ultrapure distilled water and 50% of dialysis buffer (Sebia) in a sterile container. The column was refilled to 4 mL volume with this solution and centrifuged at 1.878× *g* for 20 min. The 200 μL obtained was transferred to a 1.5 mL microcentrifuge tube and subjected to the CE. Once the electrophoretic computer program provided by the equipment manufacturer (phoresis.exe) was started, microcentrifuge tubes with the dialyzed and concentrated urine were inserted in pairs into the electrophoresis instrument. When the automatic CE process finished, the electrophoretograms appeared on the computer program interface. The final result of CE is a profile that represents the different protein fractions contained in feline urine, which can vary depending on the amount of protein excreted.

As a quality control and manufacturer recommendation, frozen aliquoted serum from a healthy cat was included, diluted in running buffer at 1:49, and migrated before every run.

**Figure 2:**
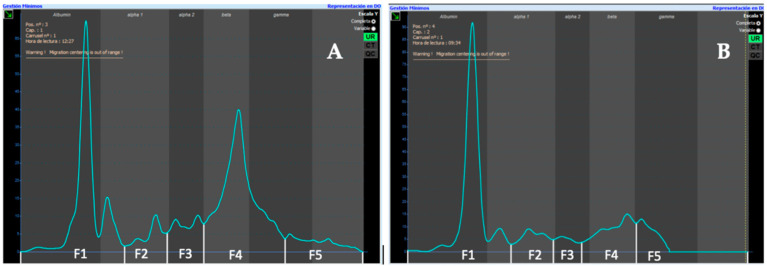
(**A**) Urinary electrophoretogram from a healthy cat performed by capillary electrophoresis. (**B**) Urinary electrophoretogram from a healthy dog performed by capillary electrophoresis.

The urinary electrophoretogram was divided into five different fractions according to serum (F1–F5). All samples were analyzed by the same person. A 1:49 diluted serum electrophoretogram from a healthy cat was placed over all urinary electrophoretograms as a guide to separate the different fractions. If necessary, protein fractions were verified and corrected by visual inspection (Figure 1). Visual comparison with urinary electrophoretograms from dogs showed similar patterns in healthy cats (Figure 2).

### 4.3. Statistical Evaluation

Statistical analysis was performed using the R commander program 3.4.3 (R Development Core Team). The Anderson–Darling test was used for each subgroup to test the hypothesis of normality. Outliers were detected by boxplot and eliminated if they were considered aberrant observations, although the emphasis was on retaining them rather than deleting them. The results were considered statistically significant if *p* < 0.05. The one-way ANOVA test was used to compare the different groups, the equality of variances was checked, and the ANOVA test was used under the hypothesis of equal variances or not. Linear regression was performed to predict the value of urinary fractions based on the value of the serum fractions.

## Figures and Tables

**Table 1:** Comparison using one-way ANOVA between the different fractions of the urinary electrophoretograms in cats (G_1_) and dogs (G_2_).

Analyte	*n*	Factor	Mean	SD	*p*-Value
F1	13 123	Cat Dog	27.18 28.77	5.62 14.10	0.43
F2	14 121	Cat Dog	5.14 8.22	2.13 2.90	0.00 *
F3	14 121	Cat Dog	8.16 8.26	3.08 2.66	0.89
F4	14 123	Cat Dog	50.00 42.89	7.27 13.02	0.01 *
F5	14 117	Cat Dog	7.80 10.61	2.71 3.55	0.00 *

*Note*. F1 corresponds to albumin; F2 corresponds to alpha_1_-globulin; F3 corresponds to alpha_2_-globulin; F4 corresponds to beta-globulin; F5 corresponds to gamma-globulin. ANOVA analysis of variance; SD: standard deviation. * Significance: *p*-value < 0.05.

**Table 2:** Linear regression performed between the different fractions of the urinary and serum electrophoretograms in cats (G_1_).

Analyte	*n*	*p*-Value
F1	14	0.28
F2	14	0.28
F3	14	0.45
F4	14	0.17
F5	14	0.10

*Note*. F1 corresponds to albumin; F2 corresponds to alpha_1_-globulin; F3 corresponds to alpha_2_-globulin; F4 corresponds to beta-globulin; F5 corresponds to gamma-globulin.

## Data Availability

The data presented in this study are available on request from the corresponding authors.
